# Supplementary material for: Laboratory validation of a simplified DNA extraction protocol followed by a portable qPCR detection of M. tuberculosis DNA suitable for point of care settings
Source: PLoS One. 2024 Dec 16;19(12):e0302345. doi: 10.1371/journal.pone.0302345 (PMC11649121; doi:10.1371/journal.pone.0302345)
Supplement: S1 Table — (PDF) [file pone.0302345.s001.pdf]

S1. Table 1. Results table for Venn diagram construction calculations.

Description: 1=positive 0= negative

Samples	Sonication (Protocol 1)	Card FTA elute (Protocol 2)	GeneXpert MTB/RIF	Culture (gold standard)
1	0	0	0	0
2	0	0	0	0
3	0	0	0	0
4	1	1	1	1
5	0	0	0	0
6	0	0	0	0
7	0	0	0	0
8	0	0	0	0
9	0	0	0	0
10	0	0	0	0
11	0	0	0	0
12	0	0	0	0
13	0	0	0	0
14	0	0	0	0
15	0	0	0	0
16	0	0	1	0
17	1	1	1	1
18	1	1	1	1
19	1	1	1	1
20	0	1	1	1
21	0	0	1	0
22	1	0	1	0
23	1	1	1	1
24	0	0	0	0
25	1	1	1	1
26	1	1	1	1

27	1	1	1	1
28	0	1	1	1
29	1	1	1	1

SCRIPT

```
library(VennDiagram)
library(readxl)

# Load the data from the Excel file as a dataframe
tb_df <-
read_xlsx("/home/cdctserver/Downloads/diagramVenn/diagramaVenn(new).xlsx", sheet
= "Planilha1")

sets_list <- list(
  "GeneXpert" = which(tb_df$GeneXpert == 1),
  "Culture" = which(tb_df$Culture == 1),
  "Q3" = which(tb_df$Q3 == 1),
  "CaseTB" = which(tb_df$CaseTB == 1)
)

# Create the Venn diagram
venn.plot <- venn.diagram(
  x = sets_list,
  #category.names = c("GeneXpert", "Culture", "Q3", "CaseTB"),
  filename = NULL,
  imagetype = "png",
  category.cex = 2,
```

```
  category.fontface = "bold",
  #category.fontfamily = "sans",
  category.names.fontsize = 20,  # Change the font size of groups
  lwd = 1,
  col = "transparent",
  cat.col = "black",
  cat.cex = 2,  # Categories font size
  #cat.fontfamily = "sans",  # Fonte padr?o ("sans")
  cat.fontface = "plain",  # Sem negrito
  fill = c("#77DD77", "#ADD8E6", "#FFFF99", "#9370DB"),
  cex = 2
)

# Gets the current working directory
getwd()

# Sets the working directory to the desired location
setwd("/home/cdctserver/Downloads/diagramVenn")

# Saves the file to the specified directory
png("venn_plot_new.png", width = 1800, height = 950)
grid.draw(venn.plot)
dev.off()  # Closes the graphics device and saves the plot as a PNG file
```
