# Supplementary material for: Laboratory validation of a simplified DNA extraction protocol followed by a portable qPCR detection of M. tuberculosis DNA suitable for point of care settings
Source: PLoS One. 2024 Dec 16;19(12):e0302345. doi: 10.1371/journal.pone.0302345 (PMC11649121; doi:10.1371/journal.pone.0302345)
Supplement: S2 Table — (PDF) [file pone.0302345.s002.pdf]

S2. Table 2. Data used to calculate the averages presented on Table 3.

Dilution	CFU		Applied Biosystems StepOne™			Q3-Plus		
10^{-1}	UNC	UNC	23.8	23.8	23.6	30.08	25.46	24.81
			23.7	23.6	23.8	25	24.68	25.68
10^{-2}	150	200	30.9	29.1	29.3	27.8	28.16	27.31
			29	29	29.3	27,14	27,72	28,11
10^{-3}	40	31	31.4	31.3	31.4	28.94	28.72	28.55
			31.4	31.6	31.6	28.9	28.8	30.86
10^{-4}	5	14	32.8	33.1	35.7	32.53	0	31.02
			32.7	33.1	32.6	32,02	32,12	33,02
10^{-5}	1	0	35.9	35.3	37.9	32.53	0	31.02
			37	35.6	34.9	32,02	32,12	33,02
