# Supplementary material for: Laboratory validation of a simplified DNA extraction protocol followed by a portable qPCR detection of M. tuberculosis DNA suitable for point of care settings
Source: PLoS One. 2024 Dec 16;19(12):e0302345. doi: 10.1371/journal.pone.0302345 (PMC11649121; doi:10.1371/journal.pone.0302345)
Supplement: S3 Table — (PDF) [file pone.0302345.s003.pdf]

S4. Table 3. Detection Probability Q3 Plus and Step One.

Q3 PLUS		
Concentration	log	ct
100000	5	33.46
10000	4	32.14
1000	3	29.29
100	2	27.95
10	1	25.74

Step One		
Concentration	log	ct
100000	5	36.1
10000	4	33.3
1000	3	31.5
100	2	29.4
10	1	23.7
