# Supplementary material for: Laboratory validation of a simplified DNA extraction protocol followed by a portable qPCR detection of M. tuberculosis DNA suitable for point of care settings
Source: PLoS One. 2024 Dec 16;19(12):e0302345. doi: 10.1371/journal.pone.0302345 (PMC11649121; doi:10.1371/journal.pone.0302345)
Supplement: S4 Table — (PDF) [file pone.0302345.s004.pdf]

**S5. Table 4. Correlation between Q3 Plus and GeneXpert Ultra semiquantitative results.**

| GeneXpert Ultra | Q3 Plus | | | |
|---|---|---|---|---|
| Results | Positive | Negative | Retest | Total |
| Negative | 7 | 30 | 16 | 53 |
| Positive trace | 1 | 3 | 2 | 6 |
| Positive very low | 1 | 1 | 3 | 5 |
| Positive low | 1 | 2 | 2 | 5 |
| Positive medium | 4 | 0 | 2 | 6 |
| Positive high | 20 | 0 | 3 | 23 |
| Total | 34 | 36 | 28 | 98 |
