# Supplementary material for: Laboratory validation of a simplified DNA extraction protocol followed by a portable qPCR detection of M. tuberculosis DNA suitable for point of care settings
Source: PLoS One. 2024 Dec 16;19(12):e0302345. doi: 10.1371/journal.pone.0302345 (PMC11649121; doi:10.1371/journal.pone.0302345)
Supplement: S5 Table — (PDF) [file pone.0302345.s005.pdf]

S6. Table 5. Results table for Venn diagram construction calculations.

Description: 1=positive 0= negative

Sample	GeneXpert Ultra	Culture	Q3	CaseTB
1	1	0	0	1
2	1	0	0	1
3	1	0	1	1
4	0	0	0	0
5	1	0	0	1
6	0	0	0	0
7	0	0	0	0
8	0	0	0	0
9	0	0	0	0
10	0	0	0	0
11	1	0	0	1
12	0	0	0	0
13	1	0	1	1
14	0	0	1	0
15	1	1	1	1
16	0	0	0	0
17	0	0	0	0
18	0	0	0	0
19	0	0	0	0
20	0	0	0	0
21	1	0	1	1
22	1	1	1	1
23	1	1	1	1
24	1	0	1	1
25	1	1	1	1
26	1	0	0	1
27	1	1	1	1
28	0	0	0	0
29	0	0	0	0
30	1	1	1	1
31	1	1	1	1
32	0	0	0	1
33	0	0	0	0
34	0	0	0	0
35	0	0	0	0
36	1	0	1	1
37	1	0	1	1
38	1	0	1	1

39	1	0	1	1
40	1	1	1	1
41	1	0	1	1
42	1	0	1	1
43	0	0	1	1
44	1	0	1	1
45	0	0	1	0
46	0	0	0	0
47	1	0	0	1
48	0	0	0	0
49	1	0	1	1
50	1	1	1	1
51	1	0	1	1
52	0	0	0	0
53	0	0	0	0
54	1	1	1	1
55	0	0	0	0
56	0	1	0	1
57	1	0	1	1
58	1	1	1	1
59	1	0	1	1
60	0	0	1	0
61	0	0	1	0
62	0	0	0	1
63	0	0	1	0
64	0	0	0	0
65	1	1	1	1
66	0	0	1	0
67	0	0	0	0
68	0	0	0	0
69	0	0	0	0
70	0	0	0	0

SCRIPT

```
library(VennDiagram)

library(readxl)


# Load the data from the Excel file as a dataframe

tb_df <- read_xlsx("/home/cdctserver/Downloads/diagramVenn/diagramaVenn(new).xlsx", sheet = "Planilha1")


sets_list <- list(

  "GeneXpert" = which(tb_df$GeneXpert == 1),

  "Culture" = which(tb_df$Culture == 1),

  "Q3" = which(tb_df$Q3 == 1),

  "CaseTB" = which(tb_df$CaseTB == 1)

)


# Create the Venn diagram

venn.plot <- venn.diagram(

  x = sets_list,

  #category.names = c("GeneXpert", "Culture", "Q3", "CaseTB"),

  filename = NULL,

  imagetype = "png",

  category.cex = 2,

  category.fontface = "bold",

  #category.fontfamily = "sans",

  category.names.fontsize = 20,  # Change the font size of groups

  lwd = 1,

  col = "transparent",

  cat.col = "black",

  cat.cex = 2,  # Categories font size

  #cat.fontfamily = "sans",  # Fonte padr?o ("sans")

  cat.fontface = "plain",  # No bold
```

```r
  fill = c("#77DD77", "#ADD8E6", "#FFFF99", "#9370DB"),
  cex = 2
)

# Gets the current working directory
getwd()

# Sets the working directory to the desired location
setwd("/home/cdctserver/Downloads/diagramVenn")

# Saves the file to the specified directory
png("venn_plot_new.png", width = 1800, height = 950)
grid.draw(venn.plot)
dev.off()  # Closes the graphics device and saves the plot as a PNG file
```
