# Supplementary material for: Laboratory validation of a simplified DNA extraction protocol followed by a portable qPCR detection of M. tuberculosis DNA suitable for point of care settings
Source: PLoS One. 2024 Dec 16;19(12):e0302345. doi: 10.1371/journal.pone.0302345 (PMC11649121; doi:10.1371/journal.pone.0302345)
Supplement: S1 Fig — (PDF) [file pone.0302345.s006.pdf]

S3. Fig 2. Plotting graphs to determine the efficiency of Q3 plus and Step one.

Description formula E = (10-1/slope – 1) x 100

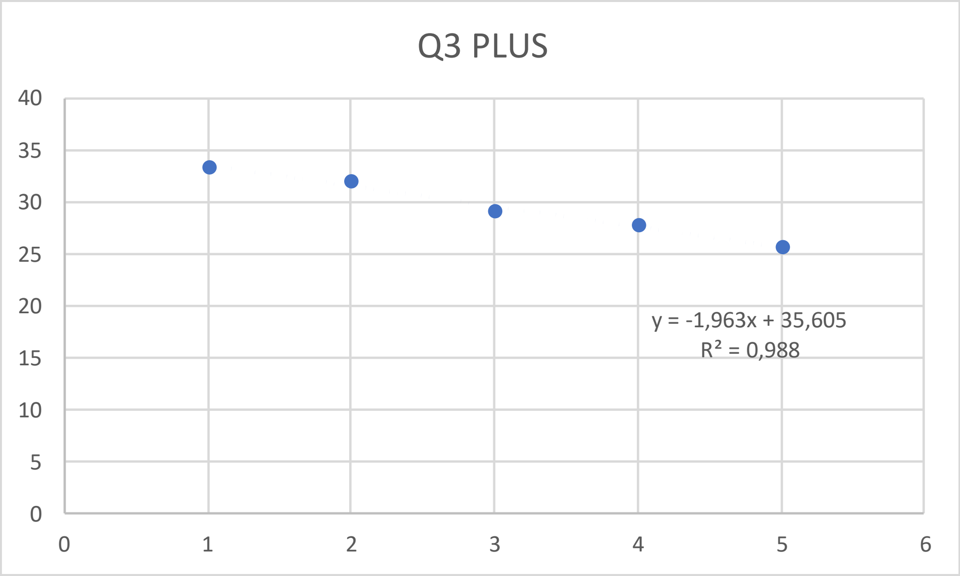

Efficiency of 216%

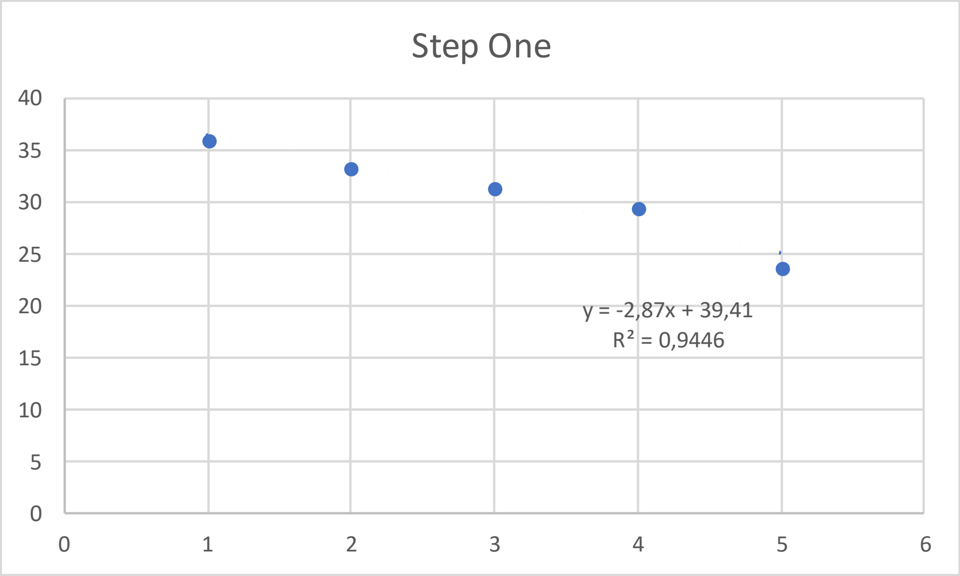

Efficiency of 118%
